# Identification of potential miRNA-mRNA regulatory network and the key miRNAs in intramuscular and subcutaneous adipose

**DOI:** 10.3389/fvets.2022.976603

**Published:** 2022-08-25

**Authors:** Hui Feng, Tianyi Liu, Salsabeel Yousuf, Xiuxiu Zhang, Wanlong Huang, Ai Li, Lingli Xie, Xiangyang Miao

**Affiliations:** State Key Laboratory of Animal Nutrition, Institute of Animal Sciences, Chinese Academy of Agricultural Sciences, Beijing, China

**Keywords:** miRNA, intramuscular fat, subcutaneous fat, pig, high-throughput sequencing

## Abstract

Intramuscular fat (IMF) is an important indicator for evaluating meat quality. Breeds with high IMF content are often accompanied by high subcutaneous fat (SCF), severely affecting the meat rate of pigs. Studying the mechanisms of miRNAs related to lipogenesis and lipid metabolism has important implications for pig breeding. We constructed two small RNA libraries from intramuscular and subcutaneous fat to evaluate the patterns of lipogenesis in Laiwu pig, a Chinese breed. A total of 286 differentially expressed miRNAs (DEmiRNAs), including 193 known miRNA and 93 novel miRNAs, were identified from two types of adipose. GO and KEGG enrichment analysis for DEmiRNAs showed that their target genes involved in many adipogenesis and lipid metabolism biological processes and signaling pathways, such as Wnt signaling pathway,MAPK signaling pathway, Hippo signaling pathway, PI3K-Akt signaling pathway, Melanogenesis, Signaling pathways regulating pluripotency of stem cells and so on. Then, we constructed a miRNA-mRNA interaction network to find out which miRNAs were the key miRNAs of regulation in Wnt signaling pathway. In this pathway, miR-331-3p, miR-339-5p, miR-874 and novel346_mature target PPARD, WNT10B, RSPO3, WNT2B. This study provides a theoretical basis for further understanding the post-transcriptional regulation mechanism of meat quality formation and predicting and treating diseases caused by ectopic fat.

## Introduction

Intramuscular fat (IMF) is one of the important factors that affect the quality of pork, however, subcutaneous fat (SCF) is considered as wastes (1). Thus, exploring how to optimize fat deposition is necessary. Besides, excessive fat deposition can lead to many diseases. Previous studies have found that SCF tissue is a relatively safe fat depot, compared with ectopic fat such as IMF and visceral fat. Increased ectopic fat deposition leads to decreased tissue function and even disease (2, 3). For instance, excessive IMF deposition cause skeletal muscle aging (4), decreasing muscle strength (5), systemic insulin resistance (6) and increasing the risk of multiple diseases, such as COVID-19 (7), rheumatoid joints (8), diabetes (9). Pigs are ideal models for exploring human diseases, with similar physiological structures to human. Understanding mechanism of adipose deposition in different tissues is a cornerstone of improving the quality of pork and exploring how to cure diseases related to ectopic fat deposition.

There is a kind of special cell called fibro-adipogenic progenitors (FAPs), with the capability of adipogenic and fibrogenic differentiation, which is the main source of IMF. Platelet-derived growth factor receptor α (also named CD140a)-positive (PDGFRA+) is the marker of FAPs (1). Many factors can affect the fat-forming ability of FAPs, such as species, FAPs location, muscle damage, microenvironment, etc. More than 90% of FAPs in mice have the capability of adipogenic differentiation, and the frequency will decrease when muscles are damaged (10), however, only 30% of FAPs in humans have fat-forming ability (11). Although both IMF and SCF belong to white fat, compared with SCF, IMF has a weaker ability to form and store lipid droplets, develops later, and has lower expression levels of genes related to adipogenic differentiation and lipid metabolism (1).

In mammals, miRNAs act within Argonaute proteins to guide repression of mRNA (12). miRNAs are regarded as biomarkers and potential therapeutic targets in many diseases (13, 14). The study of miRNA key shear enzymes shows that miRNA plays a key role in the proliferation and differentiation of adipose tissue. Drosha and Dicer in mesenchymal stem cells inhibited adipocyte differentiation, and Drosha in 3T3-L1 cells inhibited adipogenesis (15, 16). There was a negative correlation between adipogenesis and miRNA production (17). Dicer enzyme is down-regulated in adipose tissue, resulting in reduced miRNA treatment (18). Adipocyte-specific knockout of Dicer in mice resulted in severe depletion of white adipose tissue and decreased expression of fat-related genes (19). MiRNAs that promote fat differentiation include miR-21, miR-133, mir-375, and miR-143. For example, miR-21 plays a key role in obesity treatment and lipid metabolism regulation by regulating thermogenesis, white adipocyte browning, VEGF signal transduction, apoptosis, and adipogenesis biological processes and related genes (20). MiR-133 promotes the differentiation of myogenic and white adipose precursor cells into brown adipocytes (21, 22). Overexpression of miR-375 enhanced 3T3-L1 adipocyte differentiation, increased mRNA levels of induced adipogenesis, and induction of adipocyte fatty acid-binding protein (aP2) and triglyceride (TG) accumulation (23). MiR-378 specifically increased C / EBP in adipocytes α and C / EBP β transcriptional activity of gene promoter (24). Overexpression of miR-23a-5p, miR-193a-5p and miR-193b-5p enhances precursor adipocyte differentiation and adipogenesis by targeting IGF2 (25). MiR-181d down regulated ADAMTS1 and promotes adipogenesis (26). Different from the above miRNAs, miRNAs such as let-7, miR-499, miR-206, and miR-30 inhibit the proliferation and differentiation of adipocytes. In the process of adipogenesis, the expression level of let-7 increased significantly. Constructed 3T3-L1 cells within overexpressing let-7 *in vitro*, and found that let-7 prevented cloning and prolonged cell cycle (27). MiR-499 inhibits adipogenic differentiation of skeletal muscle satellite cells by targeting the expression of adipogenesis marker gene PRDM16 (28). MiR-206 inhibits Runx1 translation and inhibits the adipogenic differentiation potential of FAPs (29). MiR-26 can inhibit FBXL19, adipogenesis and precursor adipocyte differentiation (30). These results show that miRNAs play an important regulatory role in adipogenesis and metabolism, and can be used as a therapeutic target for metabolic diseases.

At present, the living standard has been greatly improved, and people's requirements for the quality and quantity of meat have also increased. Therefore, studying the molecular mechanism of IMF and SCF deposition can improve the quality of meat products to meet the needs of consumers. This study aims to explore the mechanism of adipogenesis and lipid metabolism between different adipose tissues, by constructing miRNA tissue-specific expression patterns of IMF and SCF in Laiwu pigs using RNA-Seq. Therefore, studying the mechanism of fat deposition in different parts can not only improve meat quality and promote the development of animal husbandry but also prevent or treat a series of diseases affecting human health caused by excessive fat deposition. In this study, the intramuscular and subcutaneous adipose tissues of Laiwu pigs were selected as experimental materials. The gene expression profiles of different adipose tissues were analyzed by transcriptome sequencing and bioinformatics methods, and the key candidate genes related to lipid metabolism and adipogenic differentiation were screened and identified to explore the molecular mechanism related to optimizing fat deposition.

## Materials and methods

### Total RNA extraction and quality control

The experimental animals used in this study are three Chinese breeds known as Laiwu pigs, which were 180 days, healthy, and of similar weight. All of the female pigs were raised in the same environment with standard conditions for temperature, humidity, and ventilation in Daqian farm, Shandong province. After slaughtering pigs by bloodletting, SCF were rapidly stripped from longissimus dorsi and IMF were stripped from fat located between muscle fascia in the same tissue, respectively put them into the cryopreservation tubes marked L_ PX_ 1, 2, 3 and L_ JN_ 1, 2, 3 in advance. And frozen in liquid nitrogen, and then stored at −80°C. Total RNA was extracted by mirVana™ miRNA ISOlation Kit, according to the manufacturer's instructions. Using NanoDrop2000 spectrophotometer and Aglient Bioanalyzer 2100 to measure OD260 nm/OD280nm, RNA concentration and integrity, ensure total RNA can be used for subsequent analysis.

### Small RNA library construction and sequencing

Two small RNA libraries were constructed using IMF (L_JN) and SCF (L_PX) tissues from Laiwu pigs, using Illumina TruSeq Small RNA reagent test kit. Equal amounts of total RNA from two adipose tissues of three pigs were mixed and the mixed RNA was used to construct a small RNA library. This process includes adding adapters to total RNA, reverse transcription of cDNA, PCR amplification, and electrophoresis to separate small RNA, and recovery and purification of cDNA. Using Agilent 2,100 assessed the quality of the cDNA library. Then, sequence the quality cDNA library by Illumina HiSeqTM 2,500 sequencing platform.

### Raw data quality control and small RNA annotation

It is necessary that remove joints and low-quality fragments in raw data to get clean reads for subsequent analysis. Reads with lengths of 15–41nt and not including N-base are retained. There are many kinds of small RNAs, including miRNA, tRNA (tiRNA, TRFs), rRNA, piRNA, snRNA, etc. In order to classify and annotate the small RNA in the sequencing results, clean reads were compared with the reference genome, Rfam database(31), cDNA sequence, species repeat database (32), and miRBase database (33) turn. Bowtie software was used to conduct an error-free alignment with the miRNA mature sequence in the miRBase database, and the sequence on the alignment was considered to be a known miRNA.

### Novel miRNA prediction

After the identification of known miRNAs, some sequences in clean reads that have not been annotated to miRBase may be novel miRNAs. Using miRDeep2 software to compare the FASTA file to the reference genome, the results retain the fully matched reads with a length of 18–25nt. By predicting the miRNA precursor and comparing it with the precursor information in the miRBase database, the reads on the comparison can be considered as a candidate new miRNA for quantitative analysis (33). Prediction of candidate new miRNA precursor structure by RNAfold software (34). The sequences which have a hairpin structure were considered to be novel miRNAs. Extract the mature and star sequences of predicted novel miRNAs, and count the number of novel miRNAs.

### Differentially expressed miRNA identification and target prediction

Use the DESeq2 package (35), with |log2foldchange|≥1 and *P* ≤ 0.05 as the screening conditions, to get differentially expressed miRNAs between IMF and SCF tissues. TPM (transcript per million reads) algorithm was used to calculate the expression level of the identified mature known miRNAs and novel miRNAs.

Use miRanda to predict target genes of DEmiRNAs according to score≥150 and energy ≤ -30 kcal/mol, and retain the miRNA-mRNA pairs which were differentially expressed in IMF and SCF. The predicted results of the target relationship between miRNA and mRNA were input into Cytoscape software to construct the miRNA-mRNA regulatory network.

### Gene ontology and kyoto encyclopedia of genes and genomes pathway annotation of miRNA target genes, and miRNA-protein interaction analysis

Use clusterProfiler (36) package of R Studio to annotate target genes function, including GO enrichment and KEGG pathway analysis. GO terms and KEGG pathways were considered significantly enriched when *P* ≤ 0.05. The pathway related to fat metabolism and the genes involved in this pathway were screened, and the protein-protein interaction network in this pathway was predicted by STRING database.

### Q-PCR quantitative verification

The expression level of 6 miRNAs, ssc-miR-296-5p, ssc-miR-361-3p, ssc-miR-331-3p, ssc-miR-328, sscmiR-125b, and a predicted novel miRNA novel407_mature, and 2 mRNAs, CD180 and ELF4, were randomly selected and verified by qRT-PCR. Briefly, total RNA was extracted from the IMF and SCF tissue samples with total RNA Extraction Kit (DNase I) kit (genepool, cat# gpq1801). Take 3 μL RNA was electrophoretic with 1% agarose gel to detect the integrity of RNA. miRNA cDNA Synthesis Kit (GenePool,Cat# GPQ1804) was used for reverse transcription, and BIOER LineGene 9600Plus fluorescence quantitative instrument was used to perform the PCR analysis of the miRNAs. Repeated all of the reactions in triplicate and calculate the relative quantitative expression levels by 2^−Δ*ΔCt*^ method. Incubated control miRNA gene U6 and mRNA gene GAPDH were used for the calculations of the relative expression level of the genes. Use the *T*-test to determine the significantly different expression levels.

## Results

### Evaluation of RNA-sequencing

**Figure 1:**
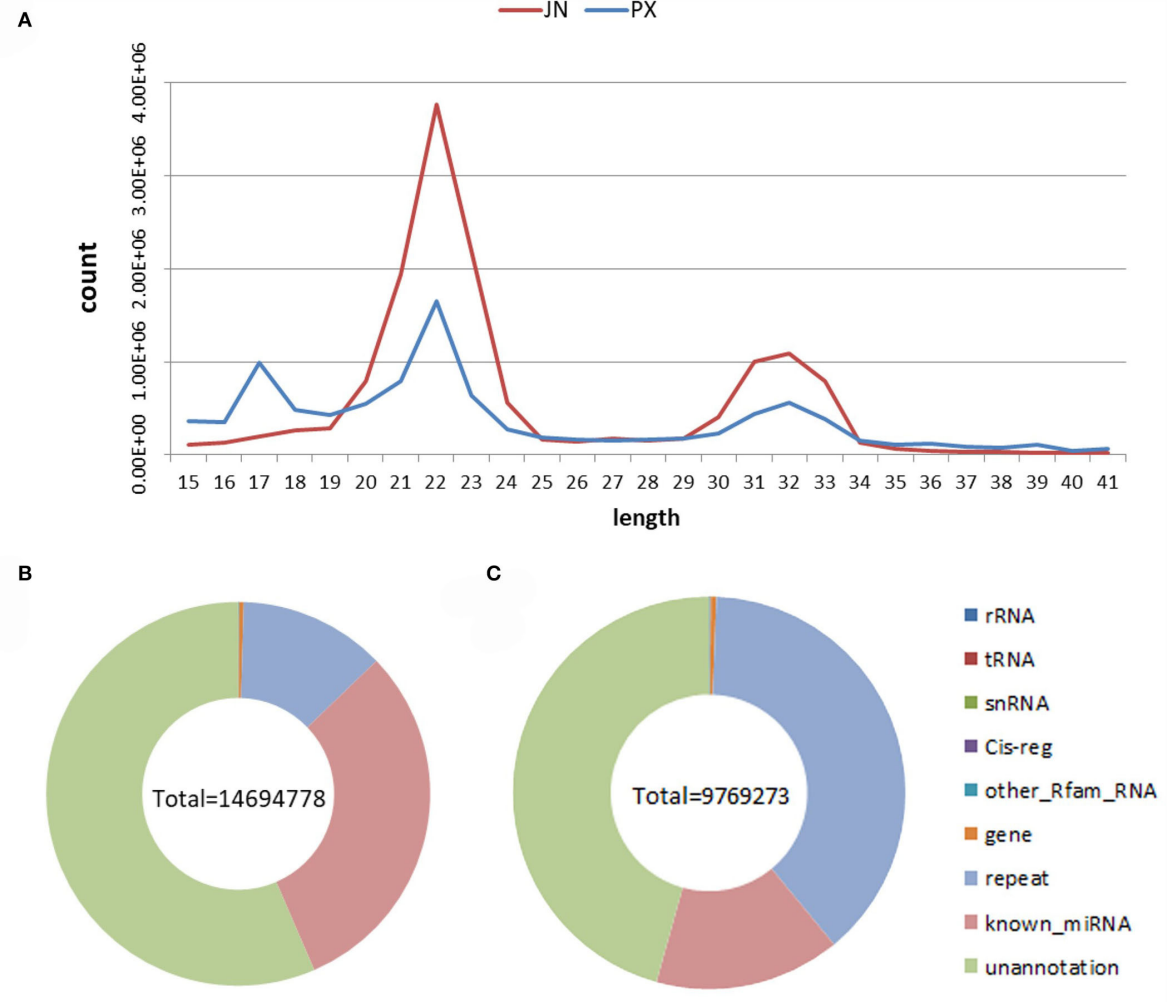
**(A)** Small RNA length distribution diagram. **(B)** L_JN; **(C)** L_PX. The type of total small RNA.

We identified 15.79 and 12.42 million raw reads, respectively from IMF and SCF miRNA libraries and obtained 14.69 and 9.77 million clean reads after removing joints and low-quality fragments. According to the statistics of the clean reads sequence length, it is found that the peak of the length distribution of these small RNA sequences is 21–25 nt, and the other peak is about 31–33 nt, which may be the distribution of piRNA, tRNA or rRNA. Of clean reads, 85.45% (12.56/14.69 million reads, IMF) and 90.79% (8.87/9.77 million reads, SCF) were mapped to the reference genome, indicating sequencing quality can be used for subsequent analysis. Rfam comparative filtering, transcript filtering, repeat comparative filtering, and miRBase database comparative annotation were performed on clean reads to obtain small RNA annotation information. As shown in Figure 1, 30.70 and 15.29% of clean reads were annotated on miRBase, and 56.49 and 45.68% of clean reads were not annotated on any database. Reads not annotated to the database were used for novel miRNA prediction.

### Identification and analysis of miRNA

**Figure 2:**
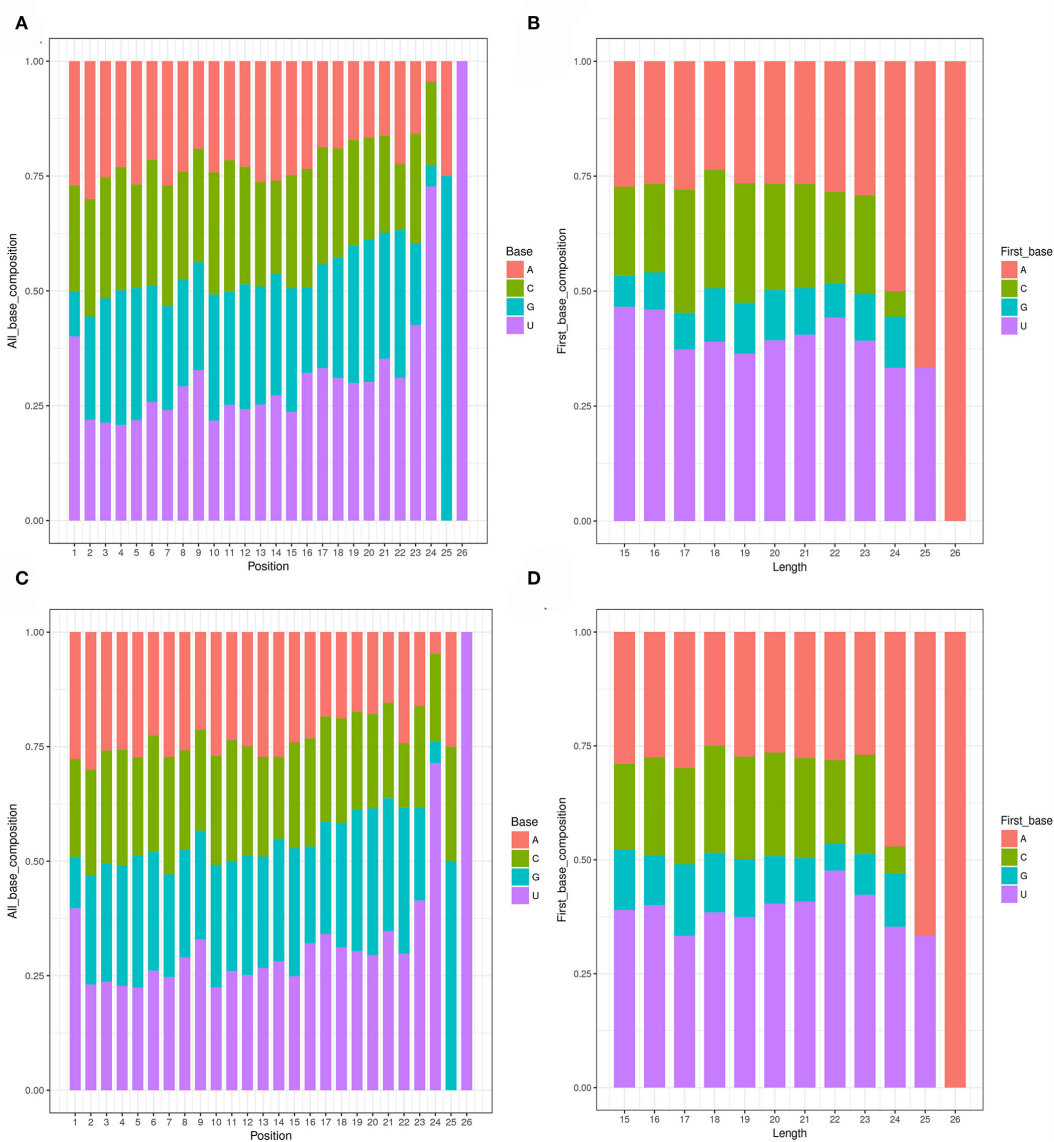
Structural characteristics of miRNAs. **(A)** Distribution map of base preference at different miRNA site in L_JN. **(B)** First base preference distribution map of miRNAs with lengths in L_JN. **(C)** Distribution map of base preference at different miRNA site in L_PX. **(D)** First base preference distribution map of miRNAs with lengths in L_PX.

After removing rRNA, tRNA, snoRNA, snRNA, and repeat sequences, 764 and 627 miRNAs were identified in IMF and SCF tissues, respectively by comparing with the miRBase database. The first base of mature miRNA has a strong preference for base U due to the specificity of the enzyme digestion site (Figures 2A,C). The tenth base is generally miRNA, which is the active site of the target gene and has a strong preference for base A (Figures 2B,D). In this study, the results of the base preference of known miRNA indicated that the sequencing results are reliable.

On the basis of their shared sequence similarity, all miRNAs identified in our study were divided into 183 families. The let-7 family contains the most kinds of miRNAs, including known miRNA such as ssc-let-7a, ssc-let-7c, ssc-let-7d-3p, ssc-mir-98 et al., and novel miRNAs such as novel403_ mature. Let-7 family is a miRNA that has been proved to play a regulatory role in adipogenesis and lipid metabolism (37), indicating that there are differences in fat deposition between the IMF and SCF tissues. In addition, the miR-10 family has the highest expression level in two adipose tissues and can regulate apoptosis and cell differentiation.

### Differential expression of miRNA

**Figure 3:**
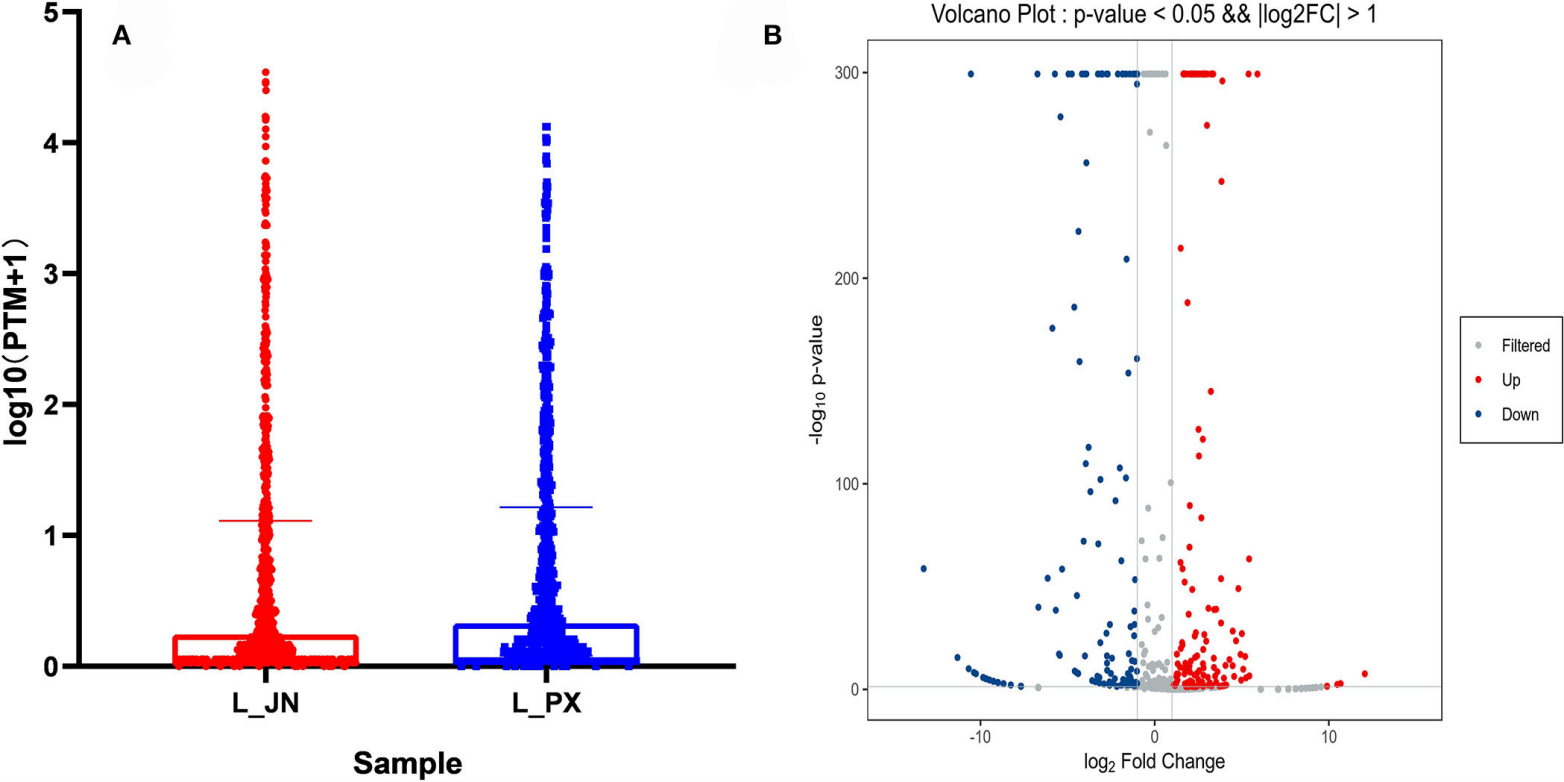
Differential expression of miRNAs. **(A)** Horizontal box diagram of miRNA expression. **(B)** Volcano map of differentially expressed miRNAs. The scatter in the figure represents the miRNAs, the black dots indicate the miRNAs with no significant differences, the red dots indicating a significant up-regulation of the miRNA, and the blue dots represent a significant down-regulation of the miRNA.

According to the diagram of miRNA expression level, it can be seen that the miRNA expression level in IMF is higher (Figure 3A). With |log2foldchange|≥1 and *P* ≤ 0.05 as the screening conditions, a total of 286 differentially expressed miRNAs (DEmiRNAs) were identified in two adipose tissues, including 193 known miRNAs and 93 novel miRNAs. Among DEmiRNAs, 146 were up-regulated and 140 were down-regulated (Figure 3B). According to the order of expression, the top 10 miRNAs were taken from IMF and SCF, respectively (Supplementary Table 2). Among them, miR-26, let-7, miR-27, and miR-1 families play important roles in adipogenic differentiation and lipid metabolism (30, 37–39).

### miRNA target genes prediction and functional enrichment analysis

**Figure 4:**
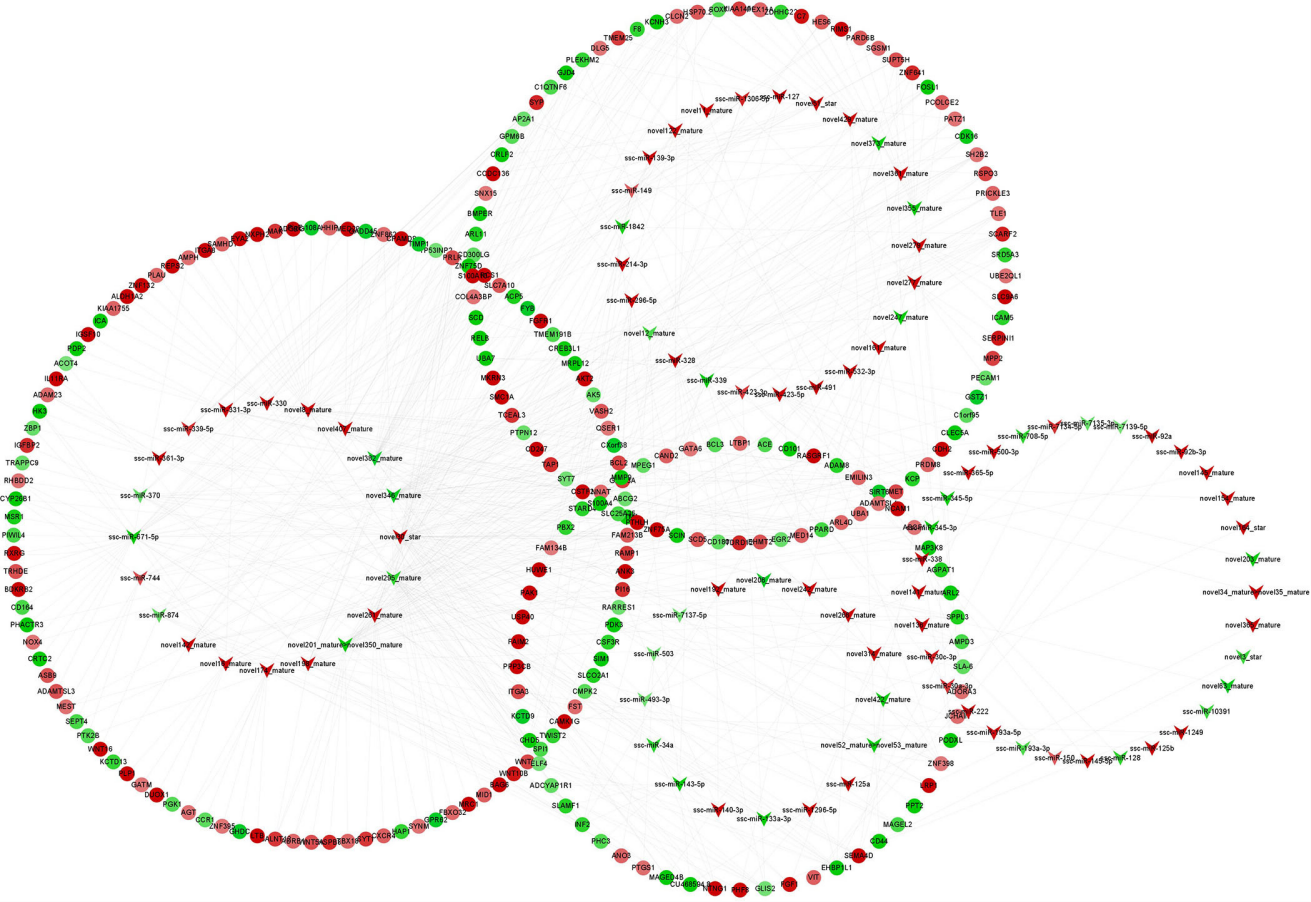
Regulatory networks of genes. miRNA-mRNA regulatory network in intramuscular and subcutaneous adipose tissue. The circle represents mRNAs, and the arrow represents miRNAs, the red represents up-regulated expression in intramuscular adipose, and the green represents down- regulated expression.

**Figure 5:**
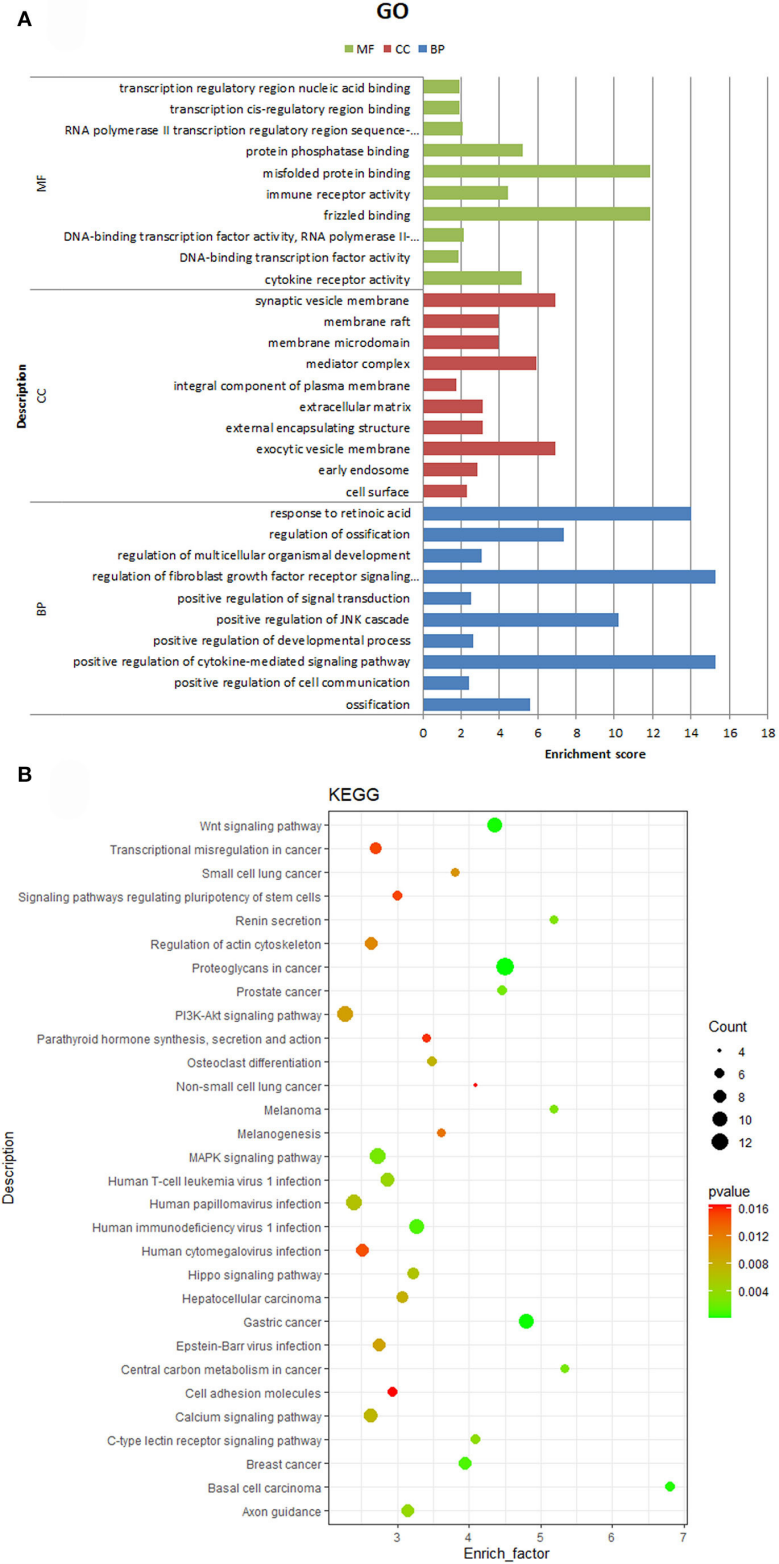
**(A)** GO enrichment analysis of target genes. The X-axis is the enrichment factor, and the Y-axis is GO term. **(B)** KEGG pathway analysis of the target genes. The X-axis is the enrichment factor, and the Y-axis is KEGG pathway.

The target genes of DEmiRNAs were predicted by miRanda software, and the differentially expressed genes (DEgenes) in IMF and SCF were screened. 93 DEmiRNAs and 616 DEgenes were involved in the regulatory network. Ssc-miR-370, ssc-miR-331-3p, ssc-miR-330, ssc-miR-361-3p, ssc-miR-744, ssc-miR-874, ssc-miR-339-5p, ssc-miR-328, and novel407_mature have more target genes (Figure 4). In order to explore the function of miRNAs, we followed the functional enrichment analysis of the target mRNAs of DEmiRNAs. The results of GO annotations show that target genes enriched in 294 GO terms, including 258 biological processes, 8 cell compositions, and 28 molecular functions. In the biological process category, target genes were involved in terms related to the regulation of cell growth and development, such as regulation of multicellular organismal development, regulation of multicellular organismal development, positive regulation of signal transduction, and regulation of fibroblast growth factor receptor signaling pathway. In the cell compositions category, target genes were involved in terms related to the structure of cell membranes, such as membrane raft, membrane microdomain, synaptic vesicle membrane, and exocytic vesicle membrane. In the molecular functions category, target genes were involved in terms related to the regulation of enzymes and transcription factor activity, such as DNA-binding transcription factor activity, RNA polymerase II-specific, cytokine receptor activity, transcription regulatory region nucleic acid binding, protein phosphatase binding, transition metal ion binding (Figure 5A). Furthermore, the predicted target genes were annotated in KEGG pathways to identify potential pathways that may be regulated by the differentially expressed miRNAs. The results indicated that target genes were enriched in 53 pathways like Wnt signaling pathway, MAPK signaling pathway, Melanoma, C-type lectin receptor signaling pathway, C-type lectin receptor signaling pathway, and PI3K-Akt signaling pathway (Figure 5B).

### The relationship between miRNAs and target genes in the Wnt signaling pathway

**Figure 6:**
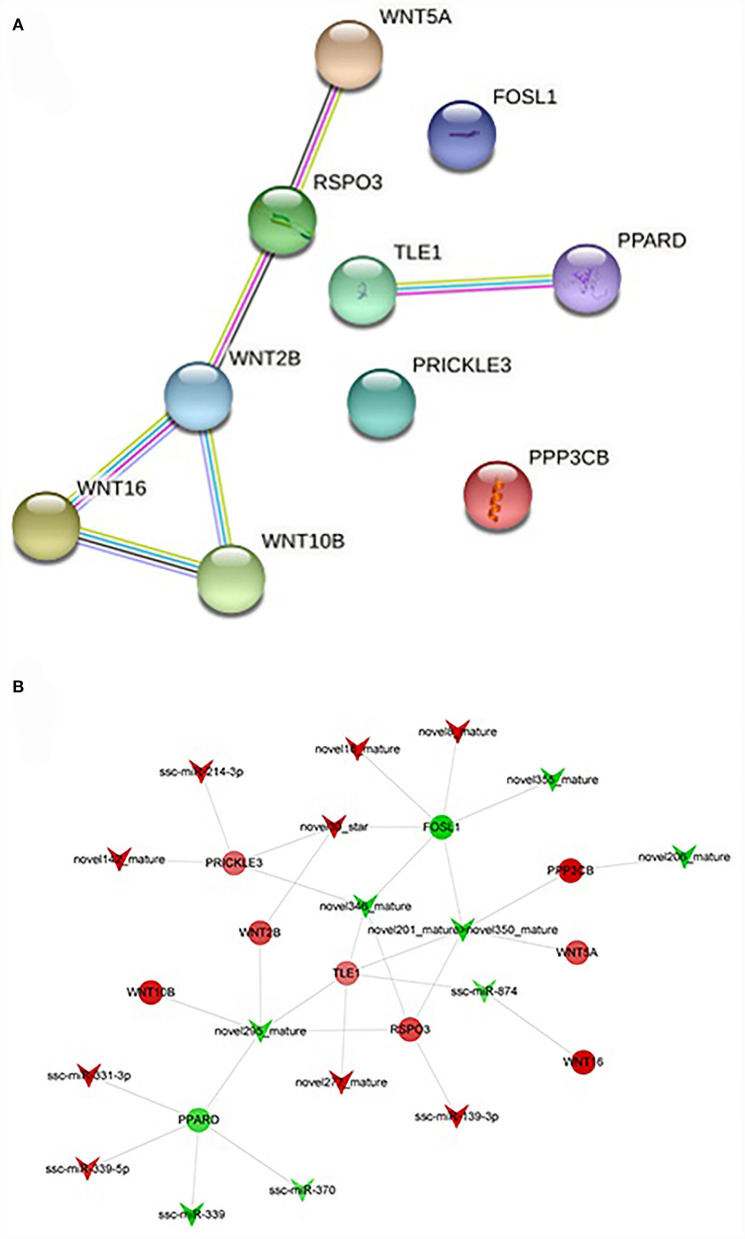
**(A)** Protein-protein interaction network of differentially expressed gengs related to Wnt signaling pathway. **(B)** The targeting relationship of genes in regulation of Wnt signaling pathway.

Wnt signaling pathway plays an important role in the process of fat deposition. We found that miRNA target genes are partially enriched in this signaling pathway, including TLE1, PRICKLE3, RSPO3, FOSL1, PPARD, WNT16, WNT5A, PPP3CB, WNT10B, and WNT2B. The query results based on string database show that there is an interactive relationship between RSPO3, WNT16, WNT5A, WNT10B, and WNT2B (Figure 6A). These target genes are regulated by 17 miRNAs regulated, such as miR-331-3p, miR-339-5p, miR-874, novel346_mature, et al (Figure 6B). These DEmiRNAs and DEgenes may cause the difference in fat deposition between IMF and SCF.

### qRT-PCR results

**Figure 7:**
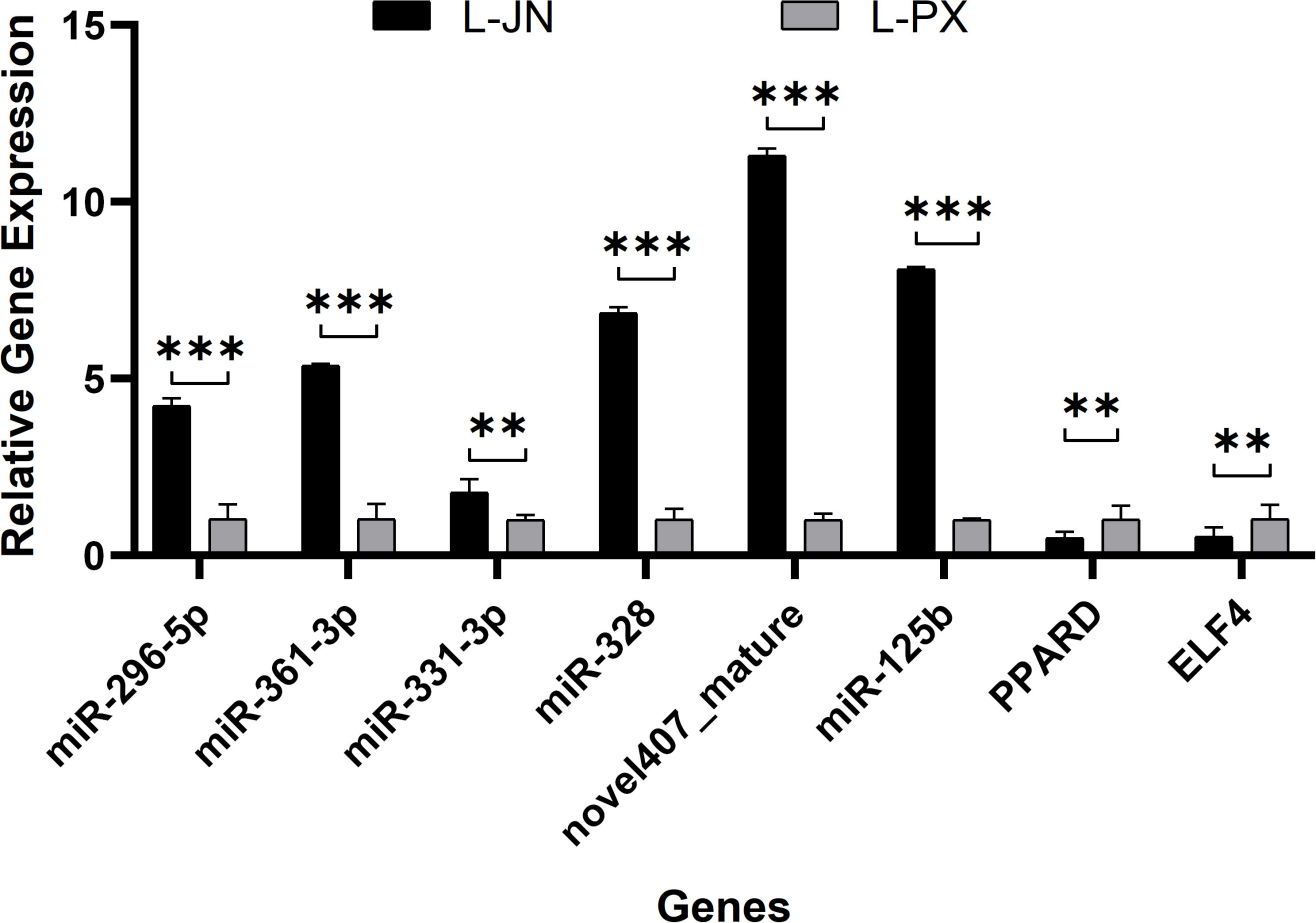
qRT-PCR verification of differentially expressed genes. The differential expression of genes between intramuscular and subcutaneous adipose tissue in Laiwu pig was verified qRT-PCR. *p < 0.05; **p < 0.01; ***p < 0.001.

The expression level of 6 differentially expressed miRNAs and 2 target genes were selected and verified by qRT-PCR (Figure 7). The results showed that 6 miRNAs (mir-296-5p, mir-361-3p, mir-331-3p, mir-328, novel407_nature, miR-125b) were downregulated, which was consistent with the results of RNA-Seq. In addition, we verified the expression of target genes (PPARD, ELF4) and proved that the sequencing results were reliable.

## Discussion

Laiwu pig is a typical local pig breed in China. It has good IMF deposition and delicious meat. We previously identified the differential expression of miRNA in fat among different breeds of pigs, cattle, and sheep, and constructed miRNA-mRNA networks (40–42). In these results, not only miRNA regulatory networks related to fat regulation were found, but also many new miRNAs were identified. It shows that the lipid deposition difference in adipose tissue between different varieties is related to the expression of miRNA, so we regulate miRNA in different parts of adipose tissue in the same species. Exploring the differential mechanism of IMF and SCF deposition and identifying the miRNA expression profile in the two adipose tissues help to improve pork quality and understanding of diseases induced by ectopic fat deposition. In this study, 286 differentially expressed miRNAs (DEmiRNAs) were identified in IMF and SCF, including 193 known miRNAs and 93 novel miRNAs. The functional annotation analysis of DEmiRNAs showed that the target gene played an important role in the process of fat differentiation, proliferation, and lipid metabolism. Among these DEmiRNAs, miR-26a has the highest expression level in IMF, which may be the main miRNA leading to the difference in fat deposition between IMF and SCF.

MiR-26a plays different roles in different stages of adipocytes, especially in the early stages of adipogenic differentiation. Overexpression of miR-26a can reduce the expression of PTEN, STK11, and other genes, inhibit PI3K-Akt signaling pathway, and promote adipocyte differentiation and lipid accumulation in the early stage of 3T3-L1 cell differentiation (43). In the early stage of adipogenic differentiation, it can also induce the expression of UCP1 and mitochondrial metabolism-related genes, and promote the metabolism of brown fat (44). Insulin can promote the expression of miR-26a, inhibit CDK5 and FOXC2, and promote the differentiation of human adipose stem cells (45). Knockout of miR-26a in mice resulted in the rapid deposition of adipose tissue in normally fed adult animals; on the contrary, overexpression of miR-26a can inhibit FBXL19 expression and adipogenesis (30). The specific expression of miR-26a in fat can moderately reduce the levels of visceral fat and lipid. In this experiment, miR-26a was highly expressed in IMF and inhibited the accumulation of lipids in IMF cells.

In this study, we constructed miRNA-mRNA regulatory networks and found that miR-331-3p, miR-339-5p, miR-874, novel346_mature et al banded with key target genes and regarded them as key miRNA in the Wnt signaling pathway. At different stages of precursor adipocyte differentiation, the expression level of miR-331-3p will increase first and then decrease. At the same time, the expression of the PPAR family will also show the same trend, and the expression level of cell differentiation-related genes will decrease, indicating that miR-331-3p can promote precursor adipocyte differentiation and inhibit proliferation (46). Fat droplets in adipocytes overexpressing miR-331-3p are larger, inhibit the expression of fatty acid metabolism-related gene DLST, and promote fatty acid synthesis (46). Other studies showed that the expression count of miR-331-3p in skeletal muscle cells was 48 (47), which was significantly lower than that in intramuscular fat, indicating that the regulation mode of miR-331-3p was also different in different cell types at the same site. Regulating the expression of ssc-miR-331-3p at different stages of adipocyte development can promote IMF deposition without increasing SCF. In our previous study, miR-874 was downregulated in bovine intramuscular fat (compared with Holstein cattle), indicating that miR-874 is a potential key miRNA for intramuscular fat regulation (40). Similarly, in the experiment comparing subcutaneous fat deposition in Chinese Huainan pigs (HN, the fat type) and Western commercial Duroc × (Landrace × Yorkshire) (DLY, the thin type) pigs, miR-874 is also at the center of regulating fat deposition network (48). These experimental results are consistent with the results of this study, indicating that miR-874 is a candidate regulator of fat deposition.

To explore the potential regulatory network of miR-331-3p in IMF, predict the target gene of miR-331-3p. Based on the differentially expressed transcriptome data of IMF and SCF in Laiwu pigs, the differential target genes of miR-331-3p in the two adipose tissues were screened for functional enrichment analysis. The results showed that miR-331-3p could significantly down-regulate SYT7, PBX2, PPARD, PDP2, ACOT4, EHBP1L1, ELF4, EGR2 et al (Figure 4). Among them, PPARD was confirmed to be a regulatory gene involved in adipocyte proliferation, differentiation, and lipid metabolism. Increasing PPARD expression in mice reduces lipids in adipose tissue and serum and increases the expression of lipid metabolism-related genes and adipogenesis and storage genes (49). PPARD agonists can promote cholesterol accumulation in macrophages, reduce obesity (50), increase fatty acid oxidation and oxidative phosphorylation in skeletal muscle, and increase insulin sensitivity (51). Therefore, PPARD agonists have been used as drug targets for the treatment of obesity and related diseases. Nucleotide polymorphism is the main factor affecting PPARD, and rs2016520 polymorphism has been proved to be closely related to human fat metabolism (52, 53). Overexpression of mutant PPARD (serine 112 mutated to alanine) and wild-type PPARD in mouse myocytes can promote adipogenesis and inhibit the expression of the myogenic marker gene MRF4, and mutant PPARD has a greater ability to promote adipogenesis (54). In the presence of PPARG ligand, porcine PPARD was overexpressed in myogenic cell C2C12, which promoted the expression of endogenous PPARG, adipogenesis, and adipogenic genes, and reduced the expression levels of myogenic protein and MRF4 (55). PPARD is involved in the biological process of adipogenesis and metabolism, which is a complex process. In the process of intramuscular adipogenesis, PPARD may not be directly involved and needs to be co-expressed with PPARG to promote the differentiation of myoblasts into adipocytes, but the specific mechanism is not clear and needs to be further discussed.

A number of novel miRNAs were identified in this study, and the target genes of novel miRNAs were significantly enriched in fat metabolism, immune response, fat metabolism-related diseases, and other signaling pathways. Among them, novel346_mature, novel295_mature, novel206_mature, novel355_mature et.al were involved in the Wnt signal pathway through banding with WNT2B, RSPO3, WNT5A, FOSL1 et.al. Wnt family genes are specifically expressed in bovine adipocytes, in which WNT2B regulates fat differentiation by activating FZD5 (56). RSPO3 promotes osteogenic differentiation of adipose stem cells through the LGR4-ERK signaling pathway (57). Subsequently, it was found that RSPO3 expression was increased in SCF, which inhibited adipogenesis and increased adipocyte apoptosis by regulating Wnt signal transduction (58). WNT5A is a fat factor that regulates the nonstandard Wnt signaling pathway and inhibits the differentiation of FAPs into adipocytes (59). FOSL1 plays an important role in suppressing adipogenesis by regulating C/EBPα, and FOSL1 transgenic mice have reduced fat deposition and the expression of fat marker genes (60). These results are consistent with the present study showing that these target genes play critical roles in intramuscular and subcutaneous fat deposition and lipid metabolism, resulting in significant differences in fat deposition between the two types of adipose tissue. In this study, we found that novel miRNAs regulate the expression levels of target genes through post-transcriptional regulation, and regulate the deposition of different fats by regulating the Wnt signaling pathway.

## Conclusion

We employed RNA-seq technology and bioinformatics tools to identify miRNAs in intramuscular and subcutaneous adipose tissue of Laiwu pig. Totally 286 differentially expressed miRNAs (193 known miRNAs and 93 novel miRNAs) were identified in the intramuscular and subcutaneous adipose tissue, including 140 down-regulated miRNAs and 146 up-regulated miRNAs. The GO annotation and KEGG enrichment analysis of target genes revealed the miRNA target genes might be involved in the Wnt signaling pathway. In the Wnt signaling pathway, 17 miRNAs (including miR-331-3p, miR-339-5p, miR-874, and novel346_mature et al) target PPARD, WNT10B, RSPO3, WNT2B, and other genes, respectively, and participate in the deposition of intramuscular fat and subcutaneous adipose tissue. In conclusion, all the results provide a theoretical basis for further revealing the deposition mechanism of intramuscular fat and subcutaneous adipose tissue and cultivating lean pigs with high meat quality, and lay a foundation for promoting the development of animal husbandry.

## Data availability statement

RNA-Seq data have been deposited in the NCBI Sequence Read Archive (SRA) database with accession numbers PRJNA870052 and PRJNA870173.

## Ethics statement

The animal study was reviewed and approved by Experimental Animal Welfare and Ethical of Institute of Animal Science, Chinese Academy of Agricultural Sciences.

## Author contributions

XM conceived, designed, performed the experiments, and wrote the paper. HF performed the experiment, data analysis, and wrote the paper. TL, SY, XZ, WH, AL, and LX performed the experiments. All authors have read and approved the final manuscript.

## Funding

This work was supported by a grant from the National Natural Science Foundation of China (No. 31970541), the Major Science and Technology Project of New Variety Breeding of Genetically Modified Organisms (Nos. 2009ZX08008-004B and 2008ZX08008–003), the Agricultural Science and Technology Innovation Program (NO. ASTIP-IAS05), and the Basic Research Fund for Central Public Research Institutes of CAAS (Y2016JC22, Y2018PT68, 2013ywf-yb-5, and 2013ywf-zd-2).

## Conflict of interest

The authors declare that the research was conducted in the absence of any commercial or financial relationships that could be construed as a potential conflict of interest.

## Publisher's note

All claims expressed in this article are solely those of the authors and do not necessarily represent those of their affiliated organizations, or those of the publisher, the editors and the reviewers. Any product that may be evaluated in this article, or claim that may be made by its manufacturer, is not guaranteed or endorsed by the publisher.

## Supplementary material

Supplementary Table 1 Gene and the corresponding primer sequences.

Supplementary Table 2 Highly expressed miRNA in IMF and SCF.

## Abbreviations

RNA-seq: RNA sequencing technology
GO: Gene ontology
KEGG: the Kyoto Encyclopedia of Genes and Genomes
IMF: Intramuscular fat
SCF: subcutaneous fat
DEmiRNAs: differentially expressed miRNAs
DEgenes: differentially expressed target genes of miRNA
PCR: polymerase chain reaction
TPM: transcript per million reads
U: Uracil
A: Adenine.
